# Dual pathways to prospective remembering

**DOI:** 10.3389/fnhum.2015.00392

**Published:** 2015-07-14

**Authors:** Mark A. McDaniel, Sharda Umanath, Gilles O. Einstein, Emily R. Waldum

**Affiliations:** ^1^Washington University in St. Louis, St. Louis, MOUSA; ^2^Furman University, Greenville, SCUSA

**Keywords:** prospective memory, spontaneous retrieval, neuroimaging of prospective memory, prospective memory paradigms, monitoring in prospective memory

## Abstract

According to the multiprocess framework (McDaniel and Einstein, 2000), the cognitive system can support prospective memory (PM) retrieval through two general pathways. One pathway depends on top–down attentional control processes that maintain activation of the intention and/or monitor the environment for the triggering or target cues that indicate that the intention should be executed. A second pathway depends on (bottom–up) spontaneous retrieval processes, processes that are often triggered by a PM target cue; critically, spontaneous retrieval is assumed not to require monitoring or active maintenance of the intention. Given demand characteristics associated with experimental settings, however, participants are often inclined to monitor, thereby potentially masking discovery of bottom–up spontaneous retrieval processes. In this article, we discuss parameters of laboratory PM paradigms to discourage monitoring and review recent behavioral evidence from such paradigms that implicate spontaneous retrieval in PM. We then re-examine the neuro-imaging evidence from the lens of the multiprocess framework and suggest some critical modifications to existing neuro-cognitive interpretations of the neuro-imaging results. These modifications illuminate possible directions and refinements for further neuro-imaging investigations of PM.

## Dual Pathways to Prospective Remembering

In the mid-1980s, Harris (1984) wrote an important review of prospective memory (PM) in which he called for further research on this “forgotten topic.” Researchers responded to his plea, and the past three decades have produced substantial theoretical and empirical development in understanding the cognitive and neurological processes involved in PM. Much of this research has examined the retrieval processes that support event-based PM, and we focus on this topic in this paper. In doing so, we highlight evidence for the existence of two fundamentally different types of retrieval processes, propose a neurocognitive model of PM processes, evaluate existing research in light of this model, and develop methodological considerations that should enable us to better isolate these retrieval processes.

A characteristic feature of PM tasks is that they do not include an explicit prompt to engage in a memory search at the time of retrieval (Craik, 1986). The high self-initiated retrieval demands of PM tasks contrast with those of explicit retrospective memory tasks like cued recall, in which the experimenter explicitly directs participants to retrieve (i.e., puts you in a retrieval mode). Thus, in an event-based PM task, such as remembering to give a friend message, there is typically no one there to put you in a retrieval mode (and to ask you to search memory for the associated action) upon encountering the friend. Instead, somehow, in the context of processing your friend as a friend (e.g., asking her how she is feeling), you have to remember to pass along the message.

## PM Retrieval Processes and Behavioral Evidence

Many researchers have proposed that this kind of retrieval can be accomplished by top–down attentional control processes that maintain activation of the intention and/or search the environment for the triggering or target cues (McDaniel and Einstein, 2000; Burgess et al., 2001; Marsh et al., 2003; Smith, 2003). These attentional control processes are generally thought to be accomplished by the frontoparietal network (Burgess et al., 2003; Cona et al., 2015) and may be conscious or unconscious (Smith et al., 2007). Moreover, they are assumed to draw on limited capacity resources, and thus when these processes are engaged, there should be detectable costs to the ongoing task.

Behavioral research has produced convincing evidence for the existence of monitoring processes and for their functional role in supporting PM. In particular, research shows that adding a PM intention often slows participants down on an ongoing task (relative to a control condition performing only the ongoing task). Smith (2003), for example, gave her participants six PM targets to learn and asked them to press a designated key whenever they later encountered any of the six targets in the context of performing a lexical decision ongoing task. Having a PM intention produced substantial slowing on the ongoing task and further, those who slowed down the most had higher PM. Researchers have typically assumed that this slowing on ongoing tasks reflects capacity consuming costs associated with top–down monitoring processes (although see Heathcote et al., 2015, for a different interpretation of the costs). Also, emphasizing the importance of the PM task (relative to the ongoing task) enhances monitoring levels or costs and also increases PM with certain types of tasks (i.e., non-focal tasks; Einstein et al., 2005).

There is now also convincing support for the existence of bottom–up spontaneous retrieval processes. By spontaneous retrieval, we mean that the occurrence of a cue can trigger retrieval of an intention in the absence of monitoring. For example, we (McDaniel et al., 2004; McDaniel and Einstein, 2007, 2011) have proposed a reflexive associative memory process that supports spontaneous retrieval. According to this view, when forming an intention (e.g., to press a designated key when seeing the word *rake*), we create an association between the target (*rake*) and action (press the “/” key) and store that in long-term memory. Later, in the absence of monitoring for the PM target, full processing of the target is likely to stimulate reflexive retrieval of the associated action and deliver it to conscious awareness. This type of retrieval might be experienced as the intention “popping” into mind, and we assume that this reflexive associative retrieval process is mediated primarily by the hippocampus and medial temporal lobes (Moscovitch, 1994).

According to the multiprocess framework, a key factor that determines whether cues are likely to trigger spontaneous retrieval of the associated intention is the extent to which the ongoing task directs relevant processing of the PM target cue (i.e., encourages focal processing). McDaniel and Einstein (2000; see Maylor, 1996 for a similar proposal) distinguished between focal processing, in which the ongoing task (i.e., at retrieval) encourages processing of the target and especially those features that were processed at encoding, and non-focal processing, in which the ongoing task does not encourage processing of those features that were processed at encoding. For example, when the ongoing task is a lexical decision task (a task that requires participants to determine whether a letter string forms a meaningful word), a PM target of a specific word (e.g., *rake*) is focal because the ongoing task directs processing of the semantic information that was likely processed at encoding. However, when the ongoing task is a lexical decision task, a PM target of a word beginning with the letter *r* is non-focal because the ongoing task does not direct processing of first letters (Scullin et al., 2010b; see Einstein and McDaniel, 2005, for additional examples of focal and non-focal processing). Following the encoding specificity principle (Tulving, 1983), the multiprocess framework assumes that spontaneous retrieval is likely (assuming the formation of a good association between the target cue and the action) when there is strong overlap between how a cue was processed at encoding and at retrieval (i.e., focal processing).

A major difficulty in testing the role of spontaneous retrieval in PM is isolating that process as the functional retrieval process. Given the demand characteristics associated with experimental settings as well as the relatively short delays (often a few minutes) between presentation of the PM instructions and the occurrence of the PM target (at least relative to many real-world delays), it seems that participants are often inclined to monitor, even with focal cues (e.g., Smith et al., 2007). Thus, if one uses conditions that induce monitoring, one could get the impression that monitoring and sustained frontoparietal activation are always necessary to accomplish PM retrieval. And this monitoring, induced by the task demands, could mask discovery of bottom–up spontaneous retrieval processes.

In order to test for and isolate spontaneous retrieval processes, one has to use task conditions that strongly discourage (and hopefully, eliminate) monitoring. One such study was conducted by Scullin et al. (2010b, Experiment 4). To test the multiprocess framework prediction that spontaneous retrieval processes can accomplish PM retrieval when the target is focal, but that monitoring processes are needed with a non-focal target, they presented participants with either a focal or non-focal PM target only once at the end of a block of over 500 lexical decision trials. Previous research had shown that monitoring declines over trials (Harrison and Einstein, 2010) and especially so when no targets are presented (Loft et al., 2008). By emphasizing the importance of the ongoing task and presenting the target after so many trials, Scullin et al. (2010b) were able to observe PM performance under conditions of no monitoring in the focal condition and little monitoring in the non-focal condition. The interesting question was whether participants’ limited abilities to sustain monitoring by the time of the occurrence of the PM targets affected focal and non-focal performance differently. Scullin et al. (2010b) found high PM performance with a focal target and very low performance with a non-focal target (the latter of which depended on whether individuals were monitoring proximal to the non-focal target). The result of high focal PM performance in the absence of monitoring suggested that spontaneous retrieval processes can support focal prospective remembering. On the other hand, very low PM performance in the non-focal condition indicated that PM retrieval with a non-focal cue is highly dependent on monitoring.

Another approach for isolating spontaneous retrieval is to present participants with a focal PM target during a phase in which the PM intention has been suspended (and thus participants are unlikely to be monitoring). For example, Scullin et al. (2009) gave participants the PM intention to press the *Q* key when they saw a particular PM cue (e.g., the word *writer*) *during an image-rating task*. After encoding this intention, the experimenters suspended the intention by telling participants that the image-rating task would occur during a later phase of the experiment and that they would not need to perform the PM task until that phase. Participants then performed a lexical decision task, during which, the PM cue *writer* was unexpectedly presented several times. The critical finding was that participants responded more slowly to *writer* than to matched-control items that occurred during the lexical decision task (i.e., the suspended phase). Importantly, because participants were told that they did not need to perform the PM task during the suspended phase, they were not devoting resources to monitoring for the PM cue. Thus, the slowed processing of that cue when it was presented during the lexical decision task reflected spontaneous noticing or retrieval of some aspects of the PM intention. This slowing to target items during a suspended phase has been found consistently (Cohen et al., 2005; Einstein et al., 2005; Marsh et al., 2006; Knight et al., 2011; Rummel et al., 2012). There is also evidence that participants spontaneously react (slow down or even produce commission errors) when processing a PM target event after they have been told that the PM task is *completed* (Scullin et al., 2012; Walser et al., 2012; Bugg and Scullin, 2013; Beck et al., 2014).

An important question in evaluating the value of a suspended intention paradigm for examining spontaneous retrieval is determining how much of the PM intention is retrieved in this type of paradigm. The observed slowing could reflect partial retrieval of the intention (perhaps general noticing or a general sense of familiarity) or full conscious retrieval of the intention. To examine this, we (Einstein et al., 2014) recently borrowed a research method from the involuntary memory literature (Kvavilashvili and Schlagman, 2011) and occasionally probed participants to tell us what they were thinking about at various points during the suspended phase. Specifically, when probed, they were asked to describe their thoughts, if any, at the moment they were stopped. Participants were always probed after neutral words, but sometimes these neutral words immediately followed a PM target cue and sometimes the neutral words were not proximal to a PM target cue. Interestingly, prior to the occurrence of the first target event, 0% (of 82 participants) reported thinking about the PM task (indicating that the suspended instructions were successful in eliminating monitoring). By contrast, when stopped after a neutral cue following the first prospective target cue, ~40% of the participants reported thinking about the PM task. Thus, it appears that PM target cues that occur in a suspended phase (when participants are not monitoring) often produce full retrieval of the PM intention.

## Usefulness of Exploring Multiple Processes

Consideration of the view that PM retrieval can be accomplished with multiple processes (McDaniel and Einstein, 2000) has the potential to refine our understanding of existing results and to give theoretical impetus for developing new insights. For example, although the general consensus is that normal aging disrupts PM, close examination of the research shows remarkable variability, with some studies showing large effects of age and others showing no or only small age effects (e.g., Kliegel et al., 2008). One interpretation of these results is that aging disrupts capacity consuming monitoring processes but tends to preserve relatively automatic retrieval processes (e.g., Craik, 1986; Cohn et al., 2008; for a different view, see Uttl, 2011). Consistent with this interpretation, Mullet et al. (2013) found no age differences on a focal task when participants relied on spontaneous retrieval processes but age differences on a non-focal task that required monitoring processes. As another example of the potential usefulness of distinguishing between monitoring and spontaneous retrieval processes, there have been interesting attempts to examine whether sleep in general, and slow wave sleep in particular, differentially affects spontaneous retrieval and monitoring (Diekelmann et al., 2013).

Our main focus in the current article is to apply this distinction to the emergent PM and neuroimaging literature. Specifically, in the following sections, we use the multiprocess framework (and related behavioral support for the framework) as a lens through which to consider the neurocognitive underpinnings of PM and refine the interpretation of the rapidly growing neuroimaging results that are being reported.

## Implications for the Neuroscience of PM

Recently, Cona et al. (2015) provided a thoughtful and ambitious integration of the neuroimaging findings related to PM. To organize these findings, they proposed a new neurocognitive model of PM, the Attention to Delayed Intention (AtoDI) Model. This model captures PM and associated neuroimaging findings as a sequential multi-phase process that begins with encoding of the intention, followed by maintenance of the intention, and concluding with intention retrieval. From the perspective of the multiprocess framework, we suggest that this overarching scheme overly simplifies the complexity of PM, and as a consequence, may draw generalities that mask important neurocognitive differences (and thus neuroimaging patterns) across various PM tasks. Specifically, our view diverges from two main characterizations that are embedded in the AtoDI model.

First, the AtoDI model characterizes PM as involving a maintenance stage in which strategic monitoring is prominent, and thereby requires significant involvement of a frontal–attentional network. Based on the multiprocess framework and the behavioral evidence discussed in the previous sections, we think it is more accurate to assume that an active maintenance stage can be present in PM (e.g., in non-focal PM tasks); however, active maintenance (and thus sustained frontal–attentional processes) is not required for PM and indeed is minimally involved, if at all, in some PM tasks (e.g., focal tasks; Einstein et al., 2005; Scullin et al., 2010a,b; see also, Gilbert et al., 2013). In the next section, we revisit the fMRI findings regarding sustained neural activity from the perspective of the multiprocess framework.

Second, the AtoDI model characterizes the retrieval stage as a spontaneous, bottom–up process (see Figure 4, Cona et al., 2015). The multiprocess framework assumes that spontaneous retrieval processes are prominent mainly when active maintenance is not present (e.g., focal cues). For PM tasks in which active maintenance is sustained, we suggest that retrieval processes are not relatively spontaneous. Instead, retrieval would likely occur in a more top–down fashion. These top–down retrieval processes might serve as a check to match the encountered cue and maintained intention with the originally encoded intention and cue (see Smith, 2003). Consequently, these PM tasks could have somewhat different, but overlapping, neural signatures than would those supported by spontaneous retrieval processes. In terms of neuroimaging results, we would expect such differences across PM tasks to appear in the transient neural activity associated with presentation of the PM cue. We develop this distinction (not evident in the AtoDI model) in a subsequent section that focuses on transient fMRI activation. (It should be noted that Cona et al., 2015, briefly suggest that PM tasks that differentially recruit strategic monitoring and spontaneous retrieval processes would likely differentially activate relevant networks, but they do not explore this in their meta-analysis.)

**FIGURE 1:**
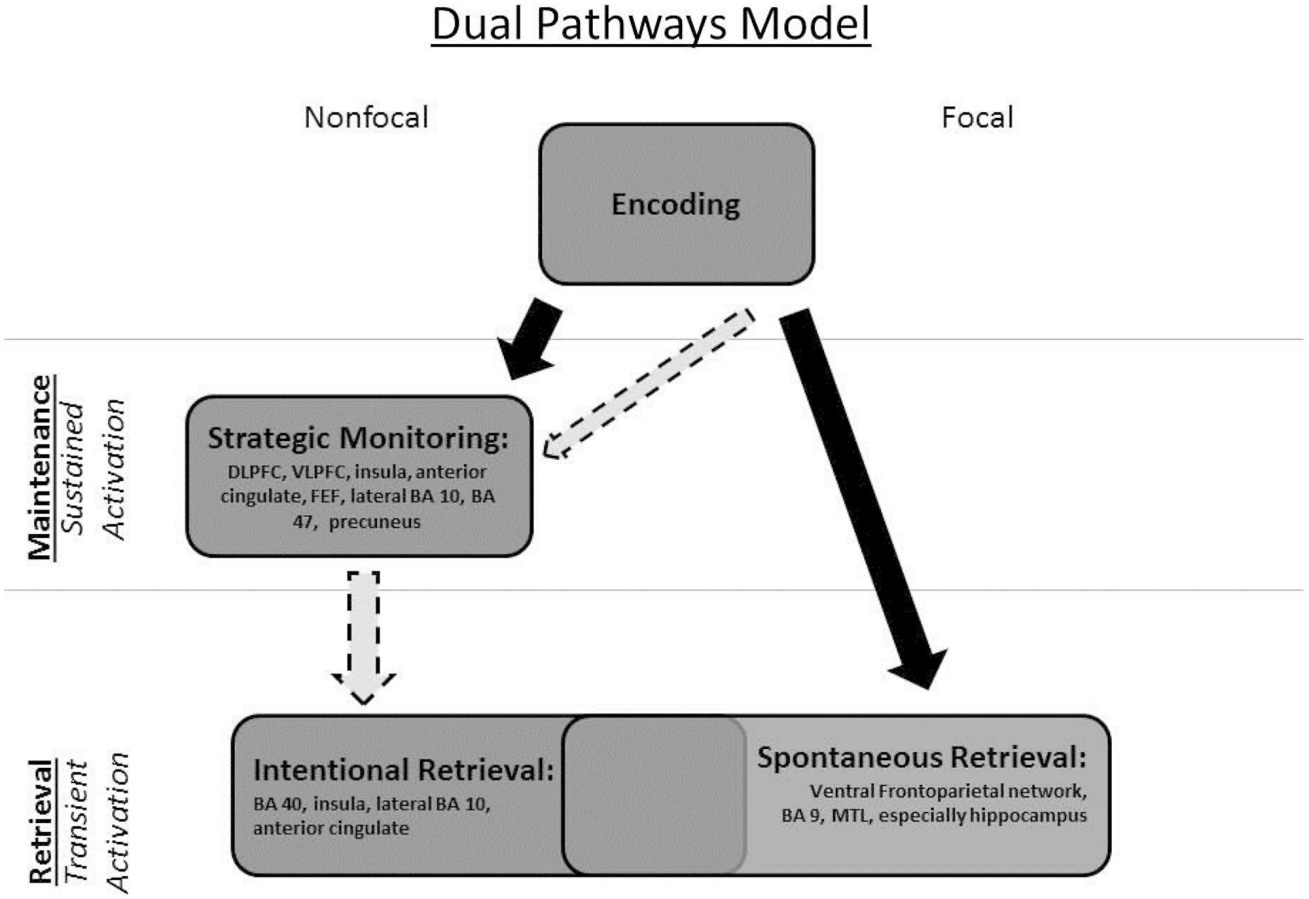
**The Dual Pathways Model of prospective memory (based on the multiprocess framework) depicted graphically.** The solid black arrows indicate the sequence of processing across stages. The dashed-line light arrows indicate that (1) even in a focal task, one can recruit strategic monitoring processes and (2) non-focal tasks may need not always involve retrieval from long-term memory.

With the above distinctions in mind, we focused on studies included in the Cona et al. (2015) meta-analysis that involved event-based PM tasks. To foreshadow, the existing neuroimaging literature bears out some of our distinctions and provides preliminary support for a modified “dual pathways” neurocognitive model of PM based on the mulitprocess framework (see **Figure 1**). The dual pathways neurocognitive model is more consistent with the multiprocess framework (see **Figure 1**) and draws attention to lingering issues regarding PM and its neural correlates. Gilbert et al. (2013)’s computational model of PM also provides support for our modified model. We emphasize at the outset that a critical limitation in the literature is that to this point, only one study has investigated the neural differences and similarities between focal and non-focal PM tasks and distinctly examined sustained versus transient neural activity: McDaniel et al. (2013). All others included only one task type or the other, with few separating sustained activity during the ongoing task and transient activity tied to the PM trials (see **Table 1**). Note that our aim is simply to draw attention to important issues in PM that can be effectively addressed through the continued examination of brain areas that are highlighted in our adjusted neurocognitive model of PM; at this stage, neuroimaging studies are not available to decisively inform our hypotheses.

**Table 1:** Neuroimaging paradigms used to investigate prospective memory and their characteristics in terms of ongoing task, focal and non-focal PM cues and examining overall, sustained, or transient activations.

Paper	Ongoing task	PM task (S/D)	PM task type	% Trials on which PM targets appear	Neural activation examined		
					Overall Only	Sustained	Transient
Okuda et al. (1998)	Repeat sets of 5 words aloud	Tap for words encoded prior to scanning (S)	Focal	5%	X		
Okuda et al. (2007)	Serial addition of digits	Squeeze hands when seven appeared (S)	Focal	12.5%	X		
Kalpouzos et al. (2010)	Virtual reality navigation of five routes	Perform list of errands (D)	Focal	N/A; 22 total		X	X
Benoit et al. (2011)	Two similar tasks: (1) navigate edges of shapes, at corners, decide a L or R turn; (2) classify capital letters for straight or curved lines	Respond when at certain corners of shapes; respond for certain letters (S)	Focal	11%		X	
Gilbert (2011)	Two-back task with words or pictures	When pic or word had colored border, encode as PM cue and key-press when see it later (S)	Focal	3%		X	X
Gilbert et al. (2012)	Same as Gilbert (2011)	Same as Gilbert (2011)	Focal	Same as Gilbert (2011)		X	X
McDaniel et al. (2013)	Semantic classification task, 525 trials	F: respond to a certain word; NF: respond to a certain syllable (S)	Focal, Non-focal	4%		X	X
Burgess et al. (2001)	Four similar tasks, e.g., press key in direction of larger of two numbers	Respond if both even numbers (S)	Non-focal	20%	X		
Burgess et al. (2003)	Three tasks from Burgess et al. (2001)	Same as Burgess et al. (2001)	Non-focal	Same as Burgess et al. (2001)	X		
den Ouden et al. (2005)	Answer questions pertaining to intentions and consequences, e.g., “you are at the cinema and have trouble seeing the screen. Do you move to another seat? Likely or unlikely?”	Respond if background is red, of five total colors (S)	Non-focal	23%	X		
Simons et al. (2006)	Two tasks: (1) Indicate the longer of a pair of a lowercase and uppercase word (2) Indicate direction of a shape relative to a triangle in a grid	Cue ID: (1) Respond to semantically associated pairs (2) Respond if shapes were a chess knight’s move away from one another (S); Intention retrieval: (1) When words are both lowercase, decide if syllables of two words are greater or less than four; (2) When shapes are the same color, decide if sides of the non-triangle are greater or less than five (S)	Non-focal	NA; 32 total	X		
Gilbert et al. (2009)	Two similar tasks: (1) Indicate direction of capital of two letters; (2) indicate direction of two dots (versus 1)	(1) Respond if same letter (2) Respond if dots form a line (S)	Non-focal	12.5%		X	X
Reynolds et al. (2009)	One-back task, respond if same or different from previous trial	Special key-press for target color of stimuli (S)	Non-focal	11%		X	X
Hashimoto et al. (2011)	Two-back task, respond if same or different from two back	Food category words (S)	Non-focal	6%		X	
Rea et al. (2011)	Decide same/diff gender of face pairs	Respond to specific face pair (S)	Non-focal	3%			X
Rusted et al. (2011)	Sort playing cards by suit	Respond to seven card (S)	Non-focal	8%			X
Beck et al. (2014)	Move joystick in the direction of a colored arrow presented on the screen in relation to a fixation cross	Key-press *instead* if arrow is red (S)	Non-focal	17%		X	X

*The studies are ordered by the type of PM cue and publication year. In the PM task, D refers to having different PM intention throughout the PM task [e.g., Kalpouzos et al. (2010), participants had a list of various errands to complete]; S refers to having the same PM intention throughout [e.g., Hashimoto et al. (2011), participants were asked to respond to every food category word].*

### Sustained Activity: Maintenance of an Intention

First, consider the sustained activity during the ongoing task, which has been assumed to reflect active maintenance of the intention and likely strategic monitoring for the PM target cue. Sustained activity related to PM processes is assessed by contrasting blocks of trials that include the PM task with control blocks that require only the ongoing task (see **Table 1** for a description of the ongoing task and PM task in each study). With PM tasks that use non-focal cues (e.g., press a key when two numbers presented on the screen are both even when the ongoing task is to indicate which has the greater value, Burgess et al., 2001; indicate when a particular face pair is presented when the ongoing task is to identify whether the faces are of the same gender, Rea et al., 2011), we expect sustained activity, reflecting Cona et al. (2015)’s proposal that the dorsal frontoparietal network is involved in strategic monitoring processes during the maintenance phase (i.e., during the ongoing task subsequent to PM encoding and prior to detection of a PM cue; see also, Reynolds et al., 2009). McDaniel et al. (2013), for instance, reported sustained activity during the ongoing processing task (a category decision task) for a non-focal PM task (execute the intention when a particular syllable, “tor,” appeared) in bilateral DLPFC (BA46), anterior cingulate, bilateral parietal lobule (BA7), bilateral precentral gyrus, and FEF (BA6). Reynolds et al. (2009) also found sustained activity for a non-focal PM task in BA 10, BA 46, and anterior cingulate cortex, as well as in bilateral inferior parietal lobe (BA 40) and the cerebellum. Additionally, Beck et al. (2014) found sustained activity for a non-focal PM task in the rostrolateral prefrontal cortex in a predefined ROI analysis.

Gilbert et al. (2009) implemented a self-initiated non-focal PM condition, in which participants were asked to indicate the side of the screen on which a capital letter appeared when presented with one letter on each side of a central fixation cross and told that they would score points for responding to particular non-focal target events (when the letters were the same, regardless of capitalization); unlike the “cued” PM condition in their study, these participants were not given further explicit instruction to complete this additional (PM) task. Presumably this condition stimulated relatively high levels of self-initiated behavior and strategic monitoring. Gilbert et al. (2009) found sustained activity in left VLPFC, left insula, bilateral supplementary motor areas, bilateral posterior cingulate, precuneus, left inferior and superior parietal cortex, right lateral temporal cortex, and left medial temporal lobe. These are likely the areas that reflect maintenance of an intention and indicate the strategic monitoring for PM cues engaged during an attention-demanding ongoing task. Generally, researchers have taken the sustained activity in the rostral-lateral area (e.g., see Burgess et al., 2003; Simons et al., 2006) and frontoparietal network (Cona et al., 2015) as requisite signatures of PM. By contrast, our claim is that not all PM tasks involve (or require) sustained activation of a rostral–lateral area or a dorsal frontoparietal network to actively maintain the PM intention and monitor for appropriate cues—that is, no active maintenance is necessary.

Specifically, we take the strong stance that for focal PM tasks that do not stimulate monitoring (see the final section for guidelines on implementing such tasks), an active maintenance stage is not involved, and accordingly, there will be little or no sustained neural activity associated with PM across the ongoing task. Supporting this prediction, McDaniel et al. (2013) found no areas showing any sustained activation (relative to control blocks without the PM task) for a focal PM task (execute the intention on presentation of a particular word during an ongoing categorization task). In a subsequent study, Beck et al. (2014) examined activation during a post-PM block, another technique to capture possible spontaneous retrieval processes. In their study, after the PM block (that included a non-focal but salient PM cue—a red colored item), participants completed another block of the ongoing task with explicit instructions that they no longer needed to perform the PM task. For this post-PM block, they found no activation in the rostrolateral prefrontal cortex from a predefined ROI analysis, though when PM targets appeared there was transient activation (discussed in the next section).

Note that the absence of sustained activation is in stark contrast to the corpus of findings with non-focal PM cues. We again emphasize, however, that important contextual features in the PM paradigm can encourage participants to actively monitor for the PM cue even when the cue is focal. For instance, when the paradigm includes many PM target cues, strategic monitoring will likely be encouraged, regardless of whether these cues are focal (see Smith, 2003). For this reason, it is important to note that it is possible that focal PM tasks may show sustained activity. Indeed, three studies using focal PM cues (Kalpouzos et al., 2010; Gilbert, 2011; Gilbert et al., 2012) have isolated sustained activity in the ongoing task from transient activity on PM trials. These areas of sustained activity included the bilateral lateral PFC (BA 10/46), medial rPFC, bilateral insula, and anterior cingulate (Gilbert, 2011), the top–down attentional network through the dorsal system (FEF and superior parietal; Kalpouzos et al., 2010), and parietal areas (Gilbert, 2011; Gilbert et al., 2012). What’s more, activation in these areas is generally greatest for subsequently successful PM. These areas are quite similar to those showing sustained activation for non-focal tasks (discussed above), including the insula, anterior cingulate, and parietal areas (Kalpouzos et al., 2010; Gilbert, 2011), suggesting that processing for these focal PM tasks involved strategic monitoring.

In light of critical features of the above paradigms, it seems likely that even with focal PM cues, participants were engaging strategic monitoring. For example, in Kalpouzos et al. (2010), participants were navigating a virtual reality environment of their hometown and had a total of 22 various focal PM tasks to perform. The ongoing task was rather minimal in that participants only needed to take a certain route as they performed the PM tasks. With such a high frequency of different targets, participants were likely steered toward monitoring for those targets (see the final section in this article), and an ongoing task that probably did not require their full attention would not discourage such strategic monitoring. In line with this supposition, the authors state that “most of the PM intentions were self-initiated, resulting in an active intention maintenance phase before target detection” (p. 7). The authors go on to state that nevertheless, “a few intentions were triggered by the perception of the targets” (p. 7).

Similarly, in Gilbert (2011), the overall procedure was specifically created such that “correct responses on PM retrieve trials had to be self-initiated on the occurrence of the appropriate item, rather than being strongly cued by the stimulus characteristics” (p. 2889), and the behavioral results converged with this claim by showing a RT cost for PM trial blocks relative to control blocks. The same procedure was used in Gilbert et al. (2012). These studies underscore that the extant PM neuroimaging literature is replete with PM tasks that stimulate strategic monitoring, even for the few studies that have used focal PM tasks. Accordingly, there has been little opportunity to reveal neural activation dynamics for the kinds of PM tasks reviewed at the outset of this article for which strategic monitoring is absent. As a consequence, we suggest that the neuroimaging literature needs to be extended to provide a more complete and accurate picture of the neural processes associated with a range of PM tasks that reflect everyday prospective remembering.

### Transient Activity: Retrieval Processes

A second point of departure from the AtoDI analysis is that the multiprocess approach anticipates that the dynamics of transient activation linked to retrieval (i.e., activation that occurs only during the target trials and not throughout the ongoing task) for focal and non-focal PM are somewhat different. This said, given that both focal and non-focal PM tasks are event-based and may both require episodic retrieval (of the encoded intention), it makes sense to expect that there would be overlap in the network of areas activated for both tasks (see for instance, Hall et al., 2014, for the neural basis of voluntary and involuntary episodic memories). However, because the retrieval process in focal PM is assumed to be spontaneous, we would expect that the retrieval processes may not involve strategic retrieval operations [associated with dorsolateral prefrontal cortex (DLPFC); Dobbins and Han, 2006; see also Hall et al., 2014], and instead may originate with bottom–up processes stimulated by hippocampal-system activation. By contrast, retrieval in non-focal PM, wherein strategic monitoring and top–down processes are involved, could involve DLPFC and activations “primed” or stimulated by aPFC structures (McDaniel et al., 2013). For example, Gilbert and colleagues [Gilbert (2011), Gilbert et al. (2013)] have proposed a model that suggests “content free” monitoring in lateral BA 10 that co-activates intention-content brain regions. Alternatively, it is possible that when the non-focal PM cue appears, there may not be a need for retrieval *per se* (from long-term memory) because the intention has been actively maintained in working memory throughout. Again, we simply draw attention to some interesting and suggestive data as very little work has directly examined this issue.

Comparisons across several studies that differentiated sustained and transient activity indicate that somewhat different areas might be involved in retrieval for focal versus non-focal PM. Transient activity related to PM related processes is determined by comparing activations during PM cue presentation to activations for non-target trials during PM blocks (Kalpouzos et al., 2010, these were trials at the end of the task) or trials during control blocks (or both, McDaniel et al., 2013). Transient activation for focal tasks was found in pre/post-central gyrus (BA3/4) and cerebellum (Gilbert, 2011) especially for successful PM (Gilbert et al., 2012). Other implicated areas include posterior cingulate (Gilbert, 2011), VLPFC and left MTL including the hippocampus (Kalpouzos et al., 2010). Additionally, medial PFC (BA9/10), caudate, and the occipito-temporal cortex showed differential activation between successful and unsuccessful PM (Gilbert et al., 2012). These findings are consistent with Cona et al. (2015)’s hypothesis that the ventral frontoparietal network may underlie spontaneous retrieval processes. It is important to be reminded that though Kalpouzos et al. (2010), Gilbert (2011), and Gilbert et al. (2012) used focal cues, they also included design features that likely stimulated strategic monitoring for the cues. Consequently, transient activations from these studies may occasionally indicate spontaneous retrieval for target trials on which monitoring may have momentarily lapsed prior to the target trial, but such transient activations are likely blended with those on trials for which monitoring was engaged. Thus, future exploration is critical for more decisive evidence regarding these areas’ involvement in spontaneous retrieval.

By contrast, transient activation results from studies using only non-focal tasks showed somewhat different patterns. Gilbert et al. (2009) found that in their self-initiated non-focal task, insula, right inferior parietal, bilateral lateral rostral PFC, bilateral ventral temporal cortex, and left lateral occipital cortex showed increased activation along with the insula and parietal areas showing increased activation for successful PM. Beck et al. (2014) found transient activation in several areas including right DLPFC, medial superior frontal gyrus, right middle inferior frontal gyrus, bilateral anterior insula, left posterior inferior frontal gyrus, left posterior medial frontal gyrus, bilateral medial temporal gyrus, bilateral ventral parietal cortex, precuneus, and posterior cingulate. Two other studies focused exclusively on retrieval or the transient activity associated with non-focal PM trials (Rea et al., 2011; Rusted et al., 2011). BA 40, including the bilateral marginal gyrus (Rea et al., 2011), inferior parietal gyrus (Rea et al., 2011), and inferior parietal lobule (Rusted et al., 2011), was activated in these studies. This pattern is consistent with retrieval-related BA 40 activation generally in non-focal tasks (bilateral ventral parietal cortex: McDaniel et al., 2013; inferior parietal cortex: Gilbert et al., 2009; post-central gyrus: Beck et al., 2014), as has been emphasized previously (Burgess et al., 2011). BA 40 has also been active in non-focal tasks where only overall activity was measured (Burgess et al., 2001, 2003; Simons et al., 2006). Cona et al. (2015) associated this region with the maintenance phase of PM (see Reynolds et al., 2009), but it appears to also be involved at retrieval, but only for non-focal tasks, not for focal tasks (except see McDaniel et al., 2013). As noted earlier, a possible interpretation is when the non-focal PM cue appears, there may not be a need for retrieval *per se* from long-term memory as the intention has been actively maintained in working memory all along. On one view of working memory, this maintenance could be realized as holding PM-related information in an active state, even if not directly in the focus of attention (Cowan, 1999). Such information could require complete activation when the target cue was presented but not require retrieval from long-term memory.

Additionally, the anterior cingulate was associated with transient activity during retrieval for non-focal tasks (Rusted et al., 2011; McDaniel et al., 2013; Beck et al., 2014). Like BA 40, this area has been discussed previously as important to PM (Burgess et al., 2011), but now, it seems that it is specifically involved in non-focal PM or tasks that use more strategic monitoring. For example, a more demanding non-focal task showed activation of the area compared to a less demanding one (Simons et al., 2006). Cona et al. (2015) proposed that this area is involved in spontaneous retrieval processes. Instead, given its involvement in non-focal tasks that hinge on strategic monitoring (e.g., Hashimoto et al., 2011), we suggest that this area plays a role when strategic monitoring is engaged and thus conflict is created between the ongoing task goals and responses and the PM goals and responses. When a PM cue is detected based on strategic monitoring, conflict may arise between continuing to perform the ongoing task and retrieval of the relevant PM intention followed by execution of the PM response, thereby giving rise to the transient activity during the target trial.

In line with this possibility, note that this area also showed *sustained* activation in non-focal tasks (Reynolds et al., 2009; McDaniel et al., 2013). The idea here is that in the presence of strategic monitoring, on both non-target items (which are included in sustained activation) and target items, the individual is always confronted with the specter of both the ongoing response and the PM response. [Prior work has found activation in the anterior cingulate gyrus for focal tasks. However, as mentioned above, based on the designs of the studies and instructions to participants, it is likely that participants did not rely on spontaneous retrieval processes and were encouraged to recruit strategic monitoring (Okuda et al., 1998, 2007). As such, the authors themselves indicate that activation was likely associated with the holding of an intention.] In sum, given the anterior cingulate’s association with conflict processing, it is not surprising that non-focal tasks might recruit it at both maintenance (non-target trials) and retrieval (target trials).

The anterior cingulate may not be as activated for focal PM cues when strategic monitoring is not encouraged (Benoit et al., 2011; Gilbert et al., 2012, with the caveat noted above in mind; but see McDaniel et al., 2013, in the next paragraph). According to our working hypothesis, for focal cues, retrieval is stimulated by hippocampal systems, and in the presence of hippocampally mediated retrieval of goal information, the frontal systems are “grabbed” or dominated by this goal, thereby reducing the conflict evident when a PM cue is detected based on strategic monitoring. Overall, of note is simply that the areas activated in the focal tasks may not completely overlap with those that show transient activity in non-focal tasks. In fact, consistent with our claim that some non-focal tasks may not even have a retrieval stage, Reynolds et al. (2009) found no transient activity tied to PM trials for a non-focal task.

Interestingly, however, McDaniel et al. (2013) did not find different areas showing transient activation tied to the PM trials: equivalent transient activation was present for both focal and non-focal PM tasks in left anterior cingulate gyrus, bilateral anterior insula (BA47), bilateral FEF (BA6), right basal ganglia, thalamus, and bilateral ventral parietal cortex (BA40). However, they also conducted connectivity analyses on correct PM trials as a stricter test of dissociable effects. They found that the anterior PFC was more strongly connected to the precuneus for non-focal trials and to the right middle temporal gyrus for focal ones, perhaps indicating differential retrieval pathways for focal and non-focal tasks. Taken together, these findings suggest that further work examining the transient activity at retrieval for tasks that require more or less strategic monitoring (and rely less or more on spontaneous processes, respectively) may be fruitful.

Beck et al. (2014) provide some initial insight into spontaneous retrieval processes. In their study, modeled after Scullin et al. (2011), after a non-focal PM block participants completed another block of the ongoing task for which they were instructed that they no longer needed to perform the PM task. Critically, PM targets still appeared during this block. The logic is that if participants no longer needed to perform the PM task, they would be unlikely to strategically monitor for PM targets; thus, any responses to former PM targets, observed in associated transient activity, would be indicative of spontaneous retrieval processes. Bilateral ventral parietal cortex (or angular gyrus), the precuneus, and posterior cingulate cortex all showed increased transient activity associated with the PM targets during this post-PM block. In combination with behavioral results that indicate spontaneous retrieval (a lack of decrement in ongoing task performance), these regions may be the first clear evidence of the areas involved in spontaneous retrieval processes. The authors point out that these areas have been previously implicated in bottom–up driven attention and in episodic memory retrieval, consistent with our model. [Though the PM cue was non-focal, it might be assumed to be salient (red color), thereby stimulating noticing.]

### Other Areas of Interest

As noted above, several relevant studies including either focal or non-focal tasks did not differentiate between sustained activity associated with the ongoing task and transient activity tied to the PM trials. Even when this was the case, there were overall differences in the neural correlates of focal and non-focal PM. Though these general patterns are less incisive, we note a few of these differences as points of investigation for future work.

One prominent area that theoretically would be aligned with spontaneous retrieval is the hippocampus (Moscovitch, 1994). Yet neuroimaging results have not clearly revealed transient hippocampal activation associated with focal PM, perhaps because without very high resolution technology, these signals are difficult to detect (see also Cona et al., 2015). However, evidence that the hippocampus plays a critical role in retrieval for focal PM has been reported in a structural MRI study that examined associations between gray matter volume and focal and non-focal PM performance (Gordon et al., 2011). In older adults, focal PM but not non-focal PM performance was positively associated with medial temporal volume, most prominently that of the hippocampus.

Cona et al. (2015) drew attention to the insula, suggesting that it is involved in spontaneous retrieval processes and bottom–up detection of cues in the environment. In contrast, based on its more common activation in non-focal tasks (Burgess et al., 2001; Simons et al., 2006; Gilbert et al., 2009; Rea et al., 2011; Beck et al., 2014) than in focal tasks (Kalpouzos et al., 2010; Gilbert, 2011), we suggest that this area may be more related to strategic monitoring processes. In the few focal tasks where the insula showed activity, as discussed above participants were encouraged to use strategic monitoring. In fact, even with a non-focal task, Simons et al. (2006) found greater insula activation for a more demanding non-focal task (see also, Gilbert et al., 2009 for greater sustained activity). Interestingly, the insula has shown both sustained and transient activation in a non-focal task (Gilbert et al., 2009; Cona et al., 2015; in contrast, see Burgess et al., 2001). Prior work has associated the insula with salience processing (Cona et al., 2015). As such, the insula may serve to help strategic monitoring for cues and increase the salience of the cue (Cona et al., 2015), hence its recruitment in non-focal tasks.

Activation of BA 9 (medial lateral pre-frontal) has been associated with focal PM (Okuda et al., 1998, 2007; Benoit et al., 2011) somewhat more often than with non-focal PM (Simons et al., 2006; Hashimoto et al., 2011; Rea et al., 2011). Interestingly, this area has been previously implicated in *involuntary* episodic retrieval (Hall et al., 2008). In addition, as mentioned above, BA 9 showed transient activation for instances of successful retrieval in a focal PM task (Gilbert et al., 2012). Thus, the involvement of BA 9 may reflect spontaneous retrieval processes underlying focal PM and warrants further investigation.

Finally, no discussion of the neural correlates of PM is complete without mention of BA 10, referred to variously as rostral prefrontal cortex, frontal pole, and the frontopolar cortex. This area has been repeatedly implicated in PM tasks, and its involvement in PM has been previously discussed (Cona et al., 2015, for a review; see Burgess et al., 2011). Of interest is that this area shows involvement in both non-focal and focal PM (Okuda et al., 1998, 2007; Burgess et al., 2001, 2003; den Ouden et al., 2005; Reynolds et al., 2009; Kalpouzos et al., 2010; Benoit et al., 2011; Gilbert, 2011; Hashimoto et al., 2011; Rea et al., 2011; Rusted et al., 2011); however, the particular activation dynamics within certain areas of BA 10 differ across non-focal and focal PM tasks. Specifically, both types of tasks show deactivation of medial BA 10 (Burgess et al., 2003; Kalpouzos et al., 2010; Benoit et al., 2011). Such deactivation is thought to be environmentally driven and to signal disengagement from external stimuli, which would be expected to occur in both focal and non-focal PM (Burgess et al., 2003; Okuda et al., 2007; Benoit et al., 2011).

While medial BA 10 deactivation is commonly observed in both focal and non-focal PM, the pattern of medial BA 10 deactivation coupled with activation of lateral BA 10 (Cona et al., 2015) has been observed exclusively in non-focal tasks (Burgess et al., 2003; den Ouden et al., 2005; Simons et al., 2006; Benoit et al., 2011; Rusted et al., 2011); for focal tasks, activation of lateral BA 10 is not commonly observed. Activation of lateral BA 10 has been discussed as reflecting the directing of attention internally and modulation of internal thoughts (Burgess et al., 2003; Simons et al., 2006; Okuda et al., 2007) and as having monitoring-specific involvement in PM (Gilbert et al., 2013). It is also implicated in tasks that have a high memory load (Simons et al., 2006; Gilbert et al., 2009; Barban et al., 2014; Cona et al., 2015). Thus, in PM tasks, it may be involved in maintenance of an intention during an ongoing task (Reynolds et al., 2009; Benoit et al., 2011) and the retrieval that follows (Gilbert et al., 2009). Consistent with such evidence, the pattern of medial BA 10 deactivation and lateral BA 10 activation is even stronger for a more self-initiated non-focal task, wherein intention retrieval demands were higher (Simons et al., 2006; see also, Gilbert et al., 2009). (As noted earlier, however, Gilbert et al., 2013, have suggested a “content free” monitoring role for lateral BA 10.) Here too, the consideration of whether a PM task involves cues that are relatively focal versus non-focal informs the involvement of BA 10 in PM.

## Directions for Future Work

As outlined in the current paper, it is clear that additional work is necessary to elucidate the neural processes involved in the different types of PM. Some of this work should aim to determine differences in sustained activation associated with maintenance of intentions and transient activity tied to PM retrieval. Using the distinction between focal and non-focal PM tasks as a means to more clearly differentiate the neural mechanisms of spontaneous retrieval processes from those of strategic monitoring is necessary. Because this type of work has been lacking, it remains unclear if somewhat distinct neural dynamics associated with different retrieval processes underlie types of PM that theoretically draw on varying processes. Moving beyond investigations of BA 10 and prefrontal involvement in PM, future work might aim to understand the contributions of other commonly implicated areas, including BA 40, the anterior cingulate cortex, BA 9, and the insula. Similarly, as Cona et al. (2015) point out, further examination of the medial temporal lobes’ contribution is needed.

Finally, given that so many varied areas can be involved in different types of PM tasks, future work should also aim to clarify the connectivity of the activated regions (Gilbert, 2011). How do these areas communicate with one another and when in the process of carrying out a delayed intention do they become involved? We posit that analyses illuminating these dynamics might provide valuable insights concerning the degree to which spontaneous retrieval processes are initiated by medial/temporal hippocampal activation, which activates (“grabs”) frontal control processes to execute the intention, whereas retrieval associated with strategic monitoring might be initiated in a more top–down fashion to activate medial temporal/hippocampal areas to confirm the intention should be executed (i.e., retrieve the encoded cue—intention association). Here, we hoped simply to draw attention to the myriad of lingering questions regarding the neural correlates of PM and offer a neurocognitive model that is borne out of a strong theoretical basis in behavioral research.

## Methodological Considerations

Further progress on these topics and in distinguishing between spontaneous retrieval and monitoring processes is critically dependent on creating experimental and neuroimaging paradigms that clearly isolate spontaneous retrieval and monitoring processes in PM tasks. As noted earlier, it is very difficult to eliminate monitoring in laboratory PM paradigms, and few studies have convincingly done so. From our perspective, the current state of affairs in PM research is not unlike that found in the retrospective memory field two decades ago. Memory researchers were positing the influences of controlled and automatic processes in retrospective memory, but could not move forward until paradigms and techniques were developed to isolate these processes in memory performance. In a similar vein, as more researchers become interested in PM, it would be valuable to clearly delineate how experimental paradigms can be constructed to isolate spontaneous retrieval processes.

In examining studies that attempt to examine spontaneous retrieval, and especially those using neuroimaging techniques, as we noted in the previous section, they often use conditions that would appear to encourage a monitoring approach to the PM task. For example, in an effort to get as many measures of retrieval as possible, researchers often give participants a number of different PM target items to learn (e.g., Kalpouzos et al., 2010). In a study examining how sleep affects spontaneous retrieval, Diekelmann et al. (2013) gave participants 20 cue-associate pairs to learn for their PM task. That is, when performing the ongoing task, they were asked to press the *space* bar whenever they saw any of the 20 cue words and to then type in the associated item. Although they used focal PM targets, it is unlikely that participants were relying on spontaneous retrieval to accomplish the PM task (their paradigm did not allow measurement of costs or monitoring). Instead, participants were probably actively trying to keep the PM cues in mind and rehearsing them during the ongoing task.

In this final section, we present suggestions (summarized in **Table 2**) for creating experimental paradigms that help isolate spontaneous retrieval. Before doing so, however, we want to emphasize the importance of measuring monitoring by including a condition (varied either within- or between-subjects) that assesses the speed and accuracy of performing the ongoing task in the absence of a PM task. To the extent that participants rely on spontaneous retrieval for prospective remembering, the speed and accuracy of ongoing task performance should be similar in the PM and control conditions.

**Table 2:** Suggestions for creating experimental paradigms that minimize monitoring and isolate spontaneous retrieval.

1. Use an event-based prospective memory task with as many of the following characteristics as possible. a. Use a single focal target cue. b. Minimize cues or demand characteristics (such as the title of the experiment) that suggest to participants that you are interested in their prospective memory. c. Emphasize the importance of the ongoing task, minimize the importance of the prospective memory task, and remind participants of the importance of the ongoing task from time to time. d. Use many trials on the ongoing task and delay the onset of the first target. Also, limit the number of occurrences of the target event. e. Do not specify the order of performing the prospective memory and ongoing task responses. That is, f. Make it clear to participants that they can perform the prospective memory response at any point after seeing the target (including several trials later). 2. Use a suspended (or completed) intention paradigm.

Previous research has shown that monitoring is reduced (and sometimes eliminated) when using a focal cue as opposed to a non-focal cue (Einstein et al., 2005; Scullin et al., 2010a) and when using one focal cue as opposed to multiple focal cues (Einstein et al., 2005; Cohen et al., 2008). Even using a single focal target event, however, does not ensure that participants will rely on spontaneous retrieval (Smith et al., 2007) unless additional conditions are implemented. In our recent research, we have found it important to also eliminate any reference to PM in our advertisement of the study (e.g., in the title of the experiment). Instead, our title focuses on the ongoing task such as our interest in measuring their accuracy and speed of making word judgments. Along with this, during the instructions, we emphasize the ongoing task, present the PM task as being only of secondary interest, and remind participants occasionally of the importance of the ongoing task (see Harrison et al., 2014, for an example of exact instructions). To further discourage monitoring, we have additionally told participants that the PM target will occur for only 5% of the participants and therefore not to worry about it (but to press the designated key if they happen to see it). Research has also shown that presenting fewer target events (Loft and Yeo, 2007) and delaying the onset of the first target event (Loft et al., 2008) discourages monitoring.

In addition, we believe that it is important to *not* specify a particular order for performing the PM response relative to the ongoing task response. Indeed, in an attempt to discourage a monitoring approach to the PM task, we tell participants that they can perform the PM response at any point after seeing the target event (i.e., before or after performing the ongoing task response or even several trials later). By contrast, some researchers ask participants to perform the PM response immediately and *prior to or instead of* performing the ongoing task response. We believe these latter instructions encourage monitoring and attention to the PM task in order to avoid the error of first performing the ongoing task response (see Ihle et al., 2013, for evidence of larger age effects on PM tasks that specify the order of performing the PM and ongoing tasks, presumably because these kinds of tasks demand more monitoring and cognitive control).

As described above, one can also use a suspended intention paradigm. Research has shown that participants do not monitor when they are asked to suspend their intention during an intervening task (Einstein et al., 2005; Scullin et al., 2009; Knight et al., 2011; Rummel et al., 2012). A possible advantage of using this type of paradigm to study spontaneous retrieval when conducting neuroimaging research is that it may yield a purer measure of retrieval in the sense that the observed activation is uncontaminated by processes involved in executing the action.

In conclusion, we have argued that different brain processes become prominent in different kinds of PM tasks. We believe that a fruitful (although admittedly challenging) avenue for future neuroimaging researchers, working within the constraints of current neuroimaging methodological techniques, is to develop procedures that allow the operation and measurement of the range of processes that appear to operate in real-world PM tasks.

## Conflict of Interest Statement

The authors declare that the research was conducted in the absence of any commercial or financial relationships that could be construed as a potential conflict of interest.
